# The Host Defense Proteome of Human and Bovine Milk

**DOI:** 10.1371/journal.pone.0019433

**Published:** 2011-04-27

**Authors:** Kasper Hettinga, Hein van Valenberg, Sacco de Vries, Sjef Boeren, Toon van Hooijdonk, Johan van Arendonk, Jacques Vervoort

**Affiliations:** 1 Dairy Science and Technology Group, Wageningen University, Wageningen, The Netherlands; 2 Laboratory of Biochemistry, Wageningen University, Wageningen, The Netherlands; 3 FrieslandCampina, Amersfoort, The Netherlands; 4 Animal Breeding and Genomics Centre, Wageningen University, Wageningen, The Netherlands; University of South Florida College of Medicine, United States of America

## Abstract

Milk is the single source of nutrients for the newborn mammal. The composition of milk of different mammals has been adapted during evolution of the species to fulfill the needs of the offspring. Milk not only provides nutrients, but it also serves as a medium for transfer of host defense components to the offspring. The host defense proteins in the milk of different mammalian species are expected to reveal signatures of evolution. The aim of this study is therefore to study the difference in the host defense proteome of human and bovine milk. We analyzed human and bovine milk using a shot-gun proteomics approach focusing on host defense-related proteins. In total, 268 proteins in human milk and 269 proteins in bovine milk were identified. Of these, 44 from human milk and 51 from bovine milk are related to the host defense system. Of these proteins, 33 were found in both species but with significantly different quantities. High concentrations of proteins involved in the mucosal immune system, immunoglobulin A, CD14, lactoferrin, and lysozyme, were present in human milk. The human newborn is known to be deficient for at least two of these proteins (immunoglobulin A and CD14). On the other hand, antimicrobial proteins (5 cathelicidins and lactoperoxidase) were abundant in bovine milk. The high concentration of lactoperoxidase is probably linked to the high amount of thiocyanate in the plant-based diet of cows. This first detailed analysis of host defense proteins in human and bovine milk is an important step in understanding the function of milk in the development of the immune system of these two mammals.

## Introduction

Milk is the single source of nutrients for the newborn mammal. The composition of milk of different mammals has been adapted during evolution of the species to fulfill the needs of the offspring. Milk not only provides nutrients, but it also serves as a medium for transfer of host defense components to the offspring. The host defense proteins in the milk of different mammalian species is expected to reveal signatures of evolution. Proteins are a major contributor to host defense components in milk [1], [2]. In humans, a positive relation between breastfeeding and health of babies has been noted from the time of the first recorded use of human-milk substitutes, going back thousands of years [3].

Because bovine milk is used as a substitute for human milk, it is important to know the differences in host defense proteins between human and bovine milk. Despite the description of several differences between human and bovine milk, there is limited knowledge on differences in the host defense proteome. A recent overview compared the human and bovine milk proteome [4]. Data were collected, however, from studies using various types of samples and analytical techniques. Data on the presence of cytokines and hormones, for example, were available only for human milk and not for bovine milk. As a result, we now only have limited knowledge on differences in host defense proteome between human and bovine milk.

To study the milk proteome, milk is usually separated into three protein fractions: caseins, serum, and milk fat globule membrane (MFGM) [5], [6]. As a start, the whole milk is separated in cream and skim milk. The cream contains the milk fat, which is present in globules. These globules consist of a triglyceride core surrounded by the MFGM, derived from the apical membrane of the milk-producing epithelial cells [7]. The protein component of the MFGM (about 1–4% of total milk protein) can be isolated from the cream. The skim milk can be centrifuged to obtain a casein pellet and a supernatant containing the serum proteins. The MFGM and serum protein fractions, which contain the low-abundance proteins from milk, can then be used for proteomic analyses.

In this study, we compared the proteomes of serum and MFGM from human and bovine milk, with the aim to determine differences in host defense proteomes. The overlap as well as the difference we found in the host defense proteomes increases our understanding of human and bovine milk. This knowledge will help to identify the proteins responsible for immunity-promoting properties of milk for the offspring.

## Results and Discussion

We identified a total of 268 proteins in human milk and 269 proteins in bovine milk, of which 147 proteins were found in both species (Table 1). We identified a larger number of proteins in milk then has been published previously. Most studies used excision of spots on 2D-gels, followed by mass-spectrometry e.g. [5], [8], [9]. With this 2D-gel method only excised spots are analyzed. With our 1D-gel method, however, we analyzed the whole gel lane and did, thus, not rely on visible protein staining. In addition, our 1D-gel method is more suitable for analyzing membrane proteins, which are ubiquitous in MFGM [10]. The same 1D-gel method, was recently used for studying the proteome of bovine milk serum [11] and bovine MFGM [12].

**Table 1:** Number of total, serum, and milk fat globule membrane (MFGM) proteins in human and bovine milk.

Proteins	Human	Bovine	Common
Total	268	269	147
Serum	222	192	105
MFGM	234	232	118

In bovine serum, we identified a total of 192 proteins. Previously, 148 proteins were identified in bovine milk serum [11]; 132 of these were also identified here. In bovine MFGM, we identified 232 proteins while in a previous study only 116 proteins were identified [12]; 95 of these were also identified here. Both comparisons show that our approach enabled us not only to identify about 90% of the already reported proteins but also to nearly double the number of identified proteins. Many of the newly identified proteins in our study were enzymes, that usually occur at low concentration. This suggest that the increase in number of identified proteins can be explained by the higher sensitivity of our method compared with previous methods.

The identified proteins were categorized according to their GO annotation (Table 2). Of all the proteins annotated, 44 proteins in human milk and 51 proteins in bovine milk were related to a host defense function. Although the total number of host defense proteins was similar in both milk samples, the predicted function of the individual proteins differed between species. Bovine milk, for example, contained a wider range of antibacterial proteins, whereas human milk contained a wider range of immunoglobulins.

**Table 2:** Number of protein functions according to GO annotation in human and bovine milk.

Function	Human	Bovine	Common
Cell wall/cell adhesion	21	17	8
Coagulation	3	7	3
Cytoskeleton	12	8	7
Enzymes	70	50	25
Host defense	44	51	33
Other	18	13	9
Protease inhibitor	12	15	8
Protein synthesis/chaperone	11	9	4
Signaling	15	19	7
Transport	48	64	39
Unknown	14	16	4

So far, we have reported qualitative differences in the proteome of human and bovine milk. For a better understanding of the biological differences between milk of these species, we also performed a quantitative analysis of the host defense proteome. For quantification, a filter-based sample preparation method was used, as this allows a more reproducible quantification compared to gel-based methods. The relative protein concentrations of host defense proteins in human and in bovine milk is shown in Table 3. Some host defense proteins were detected only with the qualitative (gel-based) method and not with the quantitative (filter-based) method (Table 3). The failure to detect certain proteins with the quantitative method is caused by its lower sensitivity compared with the qualitative method.

**Table 3:** Presence and relative concentration of host defense proteins in human and bovine milk serum and in human and bovine milk fat globule membrane (MFGM).

Gene code	Protein	Human serum	Bovine Serum	Human MFGM	Bovine MFGM
A1BG	Alpha-1B-glycoprotein	<1	<1	<1	<1
AGP/ORM1	Alpha-1-acid glycoprotein	<1	<1	<1	9*
B2M	Beta-2-microglobulin	94	61	<1	<1
C3	Complement component C3	65	121	12	26
C4A	Complement component C4A	21	<1	12*	<1
C4BPA	C4b-binding protein alpha chain	ND	<1	ND	<1
C6	Complement component C6	ND	<1	ND	ND
C7	Complement component C7	<1	<1	ND	<1
C9	Complement component C9	ND	<1	ND	<1
CAPG	Macrophage-capping protein	<1	ND	ND	ND
CATHL1	Cathelicidin-1	ND	<1	ND	189*
CATHL2	Cathelicidin-2	ND	ND	ND	122*
CATHL4	Cathelicidin-4	ND	ND	ND	13*
CATHL6	Cathelicidin-6	ND	ND	ND	88*
CATHL7	Cathelicidin-7	ND	ND	ND	<1
CD14	Monocyte differentiation antigen CD14	262*	5	146*	31
CD46	Membrane cofactor protein precursor	ND	<1	ND	15*
CD59	MAC-inhibitory protein	<1	ND	229	133
CD81	CD81 antigen	<1	<1	<1	<1
CD5L	CD5 antigen-like	ND	<1	ND	<1
CFB	Complement factor B (Fragment)	<1	<1	<1	<1
CFI	Complement factor I	<1	<1	<1	<1
CLU	Clusterin	151*	<1	672*	<1
CRISP3	Cysteine-rich secretory protein 3	ND	19*	ND	<1
CTSS	Cathepsin S	<1	ND	<1	ND
DCD	Dermicidin	102	61	151*	<1
DEFA3	Neutrophil defensin 3	ND	ND	<1	ND
ERAP1	Endoplasmic reticulum aminopeptidase 1	ND	<1	ND	<1
GLYCAM1	Glycosylation-dependent cell adhesion molecule 1	<1	3294*	11	2565*
HF1	Complement factor H	ND	<1	ND	<1
IGHA	Immunoglobulin alpha chain C region	4566*	<1	493*	<1
IGHG	Immunoglobulin gamma chain C region	127*	<1	112*	<1
IGJ	Immunoglobulin J chain	616*	<1	<1	<1
IGK	Immunoglobulin kappa chain C region	1285*	<1	<1	<1
IGKV	Immunoglobulin kappa chain C region	<1	ND	<1	ND
IGLC	Immunoglobulin lambda chain C region	115*	ND	<1	ND
IGLV	Immunoglobulin lambda chain V region	<1	ND	<1	ND
IGHM	Immunoglobulin mu chain C region	<1	220*	<1	214*
LBP	Lipopolysaccharide-binding protein precursor	ND	<1	ND	<1
LPO	Lactoperoxidase	20	161*	<1	10*
LTF	Lactoferrin	11182*	181	7045*	59
LYZ	Lysozyme C	3274*	<1	674*	<1
MFGE8	Milk fat globule-EGF factor 8	31	57	326	2663*
MUC1	Mucin-1	<1	<1	72	181
MUC4	Mucin-4	<1	ND	70*	ND
MUC15	Mucin-15	ND	<1	ND	213*
MUC16	Mucin-16	ND	<1	ND	<1
IPI00712983	Mucin-20-like	ND	<1	ND	<1
PIGR	Polymeric immunoglobulin receptor	2745*	422	215	799*
PSME2	Proteasome activator complex subunit 2	ND	ND	<1	ND
S100A8	S100 calcium-binding protein A8 (Calgranulin-A)	<1	ND	<1	<1
S100A9	S100 calcium-binding protein A9 (Calgranulin-B)	ND	ND	<1	<1
S100A12	S100 calcium-binding protein A12 (Calgranulin-C)	ND	ND	ND	<1
SAA3	Serum amyloid A protein	ND	ND	ND	<1
SCFV	Single-chain Fv	<1	ND	<1	ND
SERPINA1	Alpha-1-antitrypsin	31	21	<1	<1
SERPINA3	Alpha-1-antichymotrypsin	250*	<1	<1	<1
SERPING1	Plasma protease C1 inhibitor	<1	<1	<1	<1
SPP1	Osteopontin	762	451	42	78
TLR2	Toll-like receptor 2	ND	<1	27	31
VTN	Vibronectin	ND	ND	<1	ND
XDH	Xanthine dehydrogenase/oxidase	282	243	1084	1457

Numbers are averaged peak heights of the three most abundant peptides (arbitrary units).

* significantly higher (p<0.05).

<1: Detected with the qualitative method, but not the quantitative method

ND: Not detected using either qualitative or quantitative method

Immunoglobulins are the most abundant group of host defense proteins in human milk serum. A wider range as well as a larger amount of immunoglobulins was identified in the serum fraction of human milk compared with bovine milk (Table 3). Bovine colostrum was found to contain similar amounts of immunoglobulins as human colostrum [13]. The concentration of immunoglobulins in bovine milk declines faster after the first days of lactation than human milk [13], [14]. Our analysis showed that immunoglobulin A (IgA) was the most abundant immunoglobulin in human milk (Table 3; gene code: IGHA). In other studies, IgA was also found to be the most prominent immunoglobulin in milk [15], [16]. This relatively high IgA concentration in human milk has been linked to the absence of this immunoglobulin in the intestine of the newborn baby [16]. It is also known that already at the age of 4 days, a calf is able to produce IgA in its intestine [17], which probably explains the relatively low IgA concentration in mature bovine milk. The high concentration of polymeric immunoglobulin receptor (PIGR) found in human milk serum (Table 3) can be related to the high IgA concentration, because PIGR is used for the transcytosis of IgA from the basolateral to the apical side of epithelial cells [18].

The newborn human is also known be deficient in CD14, which is part of the Toll-like receptor (TLR)-4 complex [16]. The TLR-4 complex can detect lipopolysaccharides on gram-negative bacteria and subsequently activate the innate immune system. CD14 is, therefore, important for protection against pathogen invasion [16], [19]. CD14 has been shown to be present in human milk, with the highest concentration being found in colostrum [19]. Bovine colostrum contains similar amounts of CD14 as human colostrum [19]. Although CD14 was not detected by them in commercial bovine milk [19], we detected CD14 in unprocessed bovine milk serum and MFGM (Table 3). Absence of CD14 in the previous study may be related to heating of their milk, a treatment which we did not apply to our samples.

IgA and CD14 are important proteins for the mucosal immune system [20], [21]. Also lactoferrin (LTF) and lysozyme (LYZ) play an important role in the mucosal immune system [20], [21]. We found that the concentration of these two antibacterial proteins is much higher in human milk than in bovine milk (Table 3), which is consistent with literature [22]. LTF was relatively abundant in the MFGM fraction of human milk (Table 3), which may seem remarkable for a secreted protein. A previous study, however, found that part of the LTF in human milk was strongly bound to the MFGM membrane [23]. This finding may be related to the defense of the epithelial membrane of the mammary gland, as MFGM originates from the epithelial membrane. Additionally, the membrane-bound LTF may have a host defense function in the newborn. LTF and LYZ have been shown to be more abundant in colostrum than in mature milk for humans and bovines. The differences in their concentration in colostrum of humans and bovines is smaller than between the mature milks [22], [24]. The four proteins (IgA, CD14, LTF, and LYZ) described above are all part of the mucosal immune system. The newborn human is deficient in two of them (IgA and CD14) during infancy [16], whereas the calf is not [17]. Although the concentration of these two proteins is similar in bovine and human colostrum [14], [19], our data show a higher concentration of these components in mature human milk compared with mature bovine milk. This higher concentration in human milk may be related to differences in maturation of the immune system between babies and calves.

Clusterin is another protein that is more abundant in human milk than in bovine milk. Clusterin, a highly glycosylated protein that is also known as apolipoprotein J, is one of the most abundant proteins in the human MFGM fraction (Table 3). Although its function is not completely clear, clusterin has been linked to cell damage and apoptosis and has been shown to be overexpressed at damaged or stressed tissues and to provide a chaperone-like activity to protect other proteins against damage [8]. Milk fat globule-EGF factor 8 (MFGE8) is a protein that has a similar function as clusterin [25]. Our data shows that MFGE8 is more abundant in bovine milk than in human milk is (Table 3). MFGE8, known also as lactadherin and PAS-6/PAS-7, is a glycoprotein, like clusterin, but its function is not completely clear; however, it has been linked to cell damage and apoptosis [25], [26]. It was shown that MFGE8 plays an important role in the maintenance of intestinal epithelial homeostasis and the promotion of mucosal healing [25]. It may be an important milk protein, therefore, for protecting the intestinal tract of the newborn. This protective effect may be related to the finding that MFGE8 is a protein that links to apoptotic cells so they can be recognized by phagocytes for engulfment [26]. This effect on apoptotic cells corresponds to the finding that MFGE8 was upregulated in involuting mammary glands, where they undergo a substantial increase in the rate of epithelial cell apoptosis [27]. The presence of a high concentration of clusterin in human milk and of MFGE8 in bovine milk may thus coincide, because these proteins have a similar function.

Our results also show that bovine milk contains a large amount of glycosylation-dependent cell adhesion molecule 1 (GlyCAM1). This proteins is the most abundant host defense protein in bovine milk serum (Table 3). GlyCAM1, known also as lactophorin and PP3, consists of a diverse group of glycoproteins/glycopeptides. GlyCAM1 is a mucin-like antibacterial component expressed at the membrane of epithelial cells of the mammary gland. The active site of this membrane-bound GlyCAM1, however, is absent in the secreted form of the protein, as found in milk serum or whey [28]. It is possible, therefore, that secreted GlyCAM1 has a different function in milk compared with its function on the epithelial cell membrane [28], [29]. The soluble form of MFGE8 has been hypothesized to be involved in lubrication and protection of the intestinal tract and may have an antibacterial function in the intestinal tract [28].

**Figure 1:**
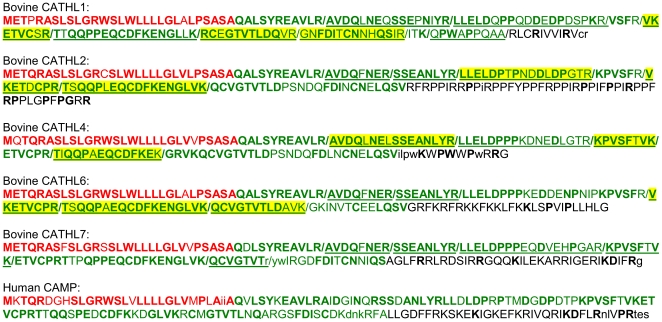
Overview of the amino acid sequence of the 5 bovine cathelicidins found. The red-colored amino acids designates the signaling peptide, the green-colored amino acids the cathelin-like domain and the black-colored amino acids the antimicrobial peptide. Bold amino acids are identical in >50% of the sequences, normal capitals show amino acids occurring in multiple sequences ad lower case amino acids occur in only one sequence. The peptides which were identified are underlined, and the yellow marking shows the peptides used for quantification. For comparison, also the amino acid sequence of the human cathelicidin is shown.

The concentration of antibacterial proteins, mainly of LTF and LYZ, was shown to be higher in human milk [22]. Our analyses revealed, however, that bovine milk contained a wider range of antibacterial proteins (Table 3). The difference in the number of antibacterial proteins was caused by 5 cathelicidins and 3 mucins, which were present only in bovine milk (Table 3). Cathelicidins are antimicrobial proteins found in different tissues of many mammals. The cathelicidin gene (gene code CAMP) has been shown to be expressed in the human mammary gland, and the polypeptide itself has been detected in ducts of the human mammary gland [30], [31]; we did, however, not detect the protein in our human milk sample. Cathelicidins have an N-terminal cathelin-like domain, which is conserved between mammals, and a diverse C-terminal antimicrobial domain (Figure 1). This antimicrobial domain differs in both length (12 to 80 residues) and structure between the different cathelicidins [32]. Most of the peptides we identified (Figure 1) were from the cathelin-like domain. Although this domain of the protein is conserved in the different cathelicidins, there are enough differences in the amino acid sequence to discriminate between the cathelicidins. This cathelin-like domain is separated from the antimicrobial domain during the maturation, which is caused by neutrophil elastase [32]. This elastase and cathelicidins are present in polymorphonuclear leukocytes, but in different granules [33], [34]. The mature forms of these antimicrobial peptides are found at mucosal surfaces and within bodily secretions [35]. The bovine genome contains at least 10 cathelicidin copies, whereas the human genome contains only one [32], [36]. The expansions in the cathelicidin gene family in the bovine genome has been hypothesized to be related to increased exposure to bacteria at the epithelial surface of the bovine mammary gland [36].

In addition to these antibacterial proteins, another antibacterial protein is lactoperoxidase (LPO), which is present in higher concentrations in both serum and MFGM of bovine milk than of human milk (Table 3). The primary function of this protein is to catalyze oxidation of certain molecules, using hydrogen peroxide, to generate reactive products with a wide antimicrobial activity [36], [37]. LPO is excreted mainly in milk and saliva [36]. The concentration of LPO in bovine milk has been shown to increase significantly in the first 5 days of lactation, reaching a plateau level after 2 weeks [37]. In milk and saliva, the main component known to be oxidized is the thiocyanate ion (SCN-) [36], [37]. The diet of the cow consists mainly of plant materials and is a good source of SCN- [38]. This SCN- can be converted by LPO into hypothiocyanate (SCNO-), which is a potent inhibitor of bacterial growth [37], [38]. In human milk, however, the limiting factor for LPO activity is its low SCN- concentration [39]. The higher concentration of LPO in bovine milk, compared with human milk, may be related, therefore, to differences in SCN- availability in the diet of the cow compared with the diet of human.

In summary, results demonstrate our ability to detect a wide range of proteins, including those from the host defense system, in human and bovine milk. Qualitative and quantitative differences were found in the milk of these two mammals. A number of antimicrobial proteins (cathelicidins, lactoperoxidase) were more abundant in bovine milk. The high concentration of lactoperoxidase is probably linked to the high amount of thiocyanate in the plant-based diet of cows. Higher concentrations of four proteins involved in the mucosal defense system (IgA, CD14, LTF, and LYZ) were found in human milk than in bovine milk. It is known that the newborn baby is deficient for two of these proteins, i.e. IgA and CD14. The concentrations of these four proteins, which are relatively similar in human and bovine colostrum, are higher in mature human milk compared to mature bovine milk. These differences in concentration between species may be related to differences in the development of the immune system of babies and calves. These results may, therefore, indicate a slower maturation of the immune system in babies than in calves. This first detailed analysis of host defense proteins in human and bovine milk is an important step in understanding the function of milk in these two mammals.

## Materials and Methods

**Figure 2:**
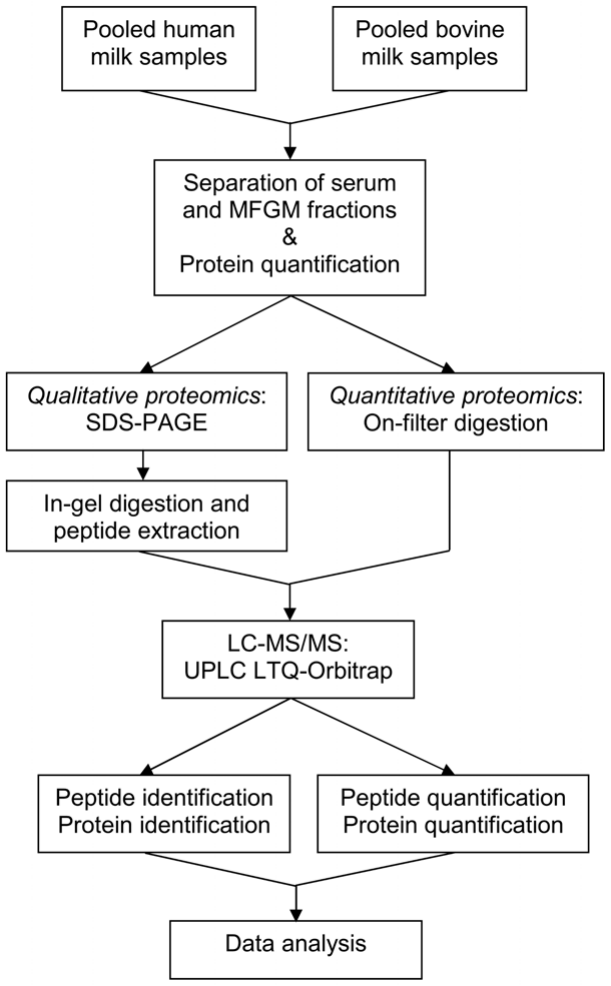
Overview of the experimental procedure.

The different steps involved in our analysis are described in this section. Figure 2 gives an overview of the experimental procedure. Milk samples were donated anonymously for this study and pooled before use, so IRB approval was not required. The regulations on which the exemption is based are 1. The “Law on medical-scientific research/Wet medisch-wetenschappelijk onderzoek” and 2. the “Code Good Practice/Code Goed Gebruik” of the “Dutch federation of Biomedical Scientific Societies”.

### Pooled milk samples

Human milk was collected from 10 healthy mothers between 3 and 10 months in lactation. Samples of 10 mL were collected and frozen for later analysis. Milk samples were donated anonymously for this study and pooled before use, so IRB approval was not required. After thawing, the 10 samples were pooled and protein fractions were separated (see below). One bovine tank milk sample was collected from the university farm “De Ossekampen” in Wageningen, The Netherlands, which was milk from 30 clinically healthy cows which were between 3 weeks and 10 months in lactation.

### Separation of milk serum and MFGM protein fractions

The separation of the serum and MFGM proteins was done as described by [5]. Milk samples were centrifuged at 1500 g for 20 min at 4°C. The cream was used for MFGM protein isolation. 5 mL of the skimmed milk was centrifuged for 90 min at 100,000 g to pellet the casein; the resulting supernatant, containing the serum proteins, was frozen at −45°C. The cream (about 10 mL) was washed 4 times by careful shaking with 30 mL phosphate-buffered saline followed by centrifugation. The washed cream was mixed 1∶1 (vol) with Milli-Q water, sonificated for 2 min, and centrifuged to remove fat. The watery subnatant, containing the MFGM proteins, was frozen at −45°C.

### Protein quantification

The protein content of all samples was quantified using a BSA Protein Assay kit (Thermo, San Jose, CA, USA). The results from these analyses were used to load the same amount of protein per fraction on the SDS-PAGE gel or centrifugal filter device.

### SDS-PAGE

Pre-cast 12% Precise Protein Gels were used with HEPES buffer (Thermo, San Jose, CA, USA). The thawed protein samples were mixed 1∶1 (vol) with 2x sample buffer (125 mM Tris-HCl (pH 6.8), 4% SDS, 20% glycerol, and 0.01% bromophenol blue in Milli-Q water; just before use, 5% β-mercaptoetanol was added) and heated for 5 min at 95°C. The protein load on the gel was about 30 µg of protein per well. The gel was run for 45 min at 130 Volt. The proteins were stained for 4 h using the Colloidal Blue Staining Kit (LC6025, Invitrogen, Carlsbad, CA, USA), and destained overnight in Milli-Q water.

### Qualitative proteomics

Except when stated otherwise, all solutions were prepared in 50 mM NH4HCO3 (pH 8). After each step, samples were sonicated for 1 min followed by spin down using a centrifuge. For each sample put on the SDS-PAGE gel, the gel lane was cut in 8 slices of about equal size. Each slice was cut into <1 mm3 pieces and transferred to low-binding microcentrifuge tubes (0030 108.094, Eppendorf, Hamburg, Germany). The gel pieces were washed twice with water. The proteins were reduced by incubation in 50 mM dithiotreitol for 1 h at 60°C followed by incubation in 100 mM iodoacetamide for 1 h at room temperature in the dark. After reduction, the gel pieces were washed 3 times with 50 mM NH4HCO3. Sample were then frozen and thawed 3 times to increase the accessibility for trypsin. 20 µL of freshly prepared trypsin solution (10 ng/µL) was added to the gel pieces. Extra 50 mM NH4HCO3 was added to cover the gel pieces completely. The gel pieces were incubated overnight at room temperature. After trypsin digestion, the supernatant was transferred to a clean low-binding microcentrifuge tube (Eppendorf). 10 µL 5% trifluoroacetic acid (TFA) in water was added to the gel pieces, and after sonication the acidic supernatant was added to the same microcentrifuge tube. 10 µL 10% acetonitrile/1% TFA was then added to the gel pieces, and after sonication the supernatant was added to the same microcentrifuge tube. The pH of the final peptide mixture was verified to be about 2, using pH paper.

### Quantitative proteomics

The previously prepared milk serum and MFGM protein fractions were analyzed in fivefold using an adapted version of [40]. 20 µL of protein solution, containing about 25 µg of protein, was solubilized in 180 µL of Solution A (100 mM Tris/HCl (pH 7.6) containing 4% SDS and 0.1 M DTT). Samples were heated for 5 min at 95°C. After cooling each sample to room temperature, 10 µL was loaded on a filter-containing centrifugal device (10–20 kDa cutoff, OD003C34; Pall, Washington, NY, USA) and centrifuged at 20,000 g for 1 min. 100 µL of Solution B (8 M Urea in 100 mM Tris/HCl pH 8) was added and the device was centrifuged for 30 min at 20,000 g. 100 µL of Solution C (0.05 M iodoacetamide in Solution B) was added. The device was mixed for 1 min, followed by incubation for 10 min. The device was then centrifuged for 30 minutes at 20,000 g. Three wash steps, with 110, 120 and 130 µL respectively, of Solution B were performed with centrifugation for 30 min at 20,000 g after each wash step. 140 µL of solution D (0.05 M NH_4_HCO_3_) was added followed by centrifugation at 20,000 g for 30 min. The filter unit was then transferred to a low-binding microcentrifuge tube (Eppendorf) and 1 µL sequencing-grade trypsin (Roche, Penzburg, Germany) in Solution D (total volume 100 µL) was added to the filter. The filters were incubated overnight at room temperature. Filters were then centrifuged for 30 min at 20,000 g. Finally, 3.5 µL 10% TFA in water was added. The pH of the final peptide mixture was verified to be about 2, using pH paper.

### LC-MS/MS

Samples were analyzed by injecting 18 µL of sample over a 0.1032 mm Prontosil 300-3-C18H (Bischoff, Germany) pre-concentration column (prepared in house) at a maximum pressure of 270 bar. Peptides were eluted from the pre-concentration column onto a 0.10200 mm Prontosil 300-3-C18H analytical column with an acetonitrile gradient at a flow of 0.5 µL/min. The gradient consisted of an increase from 9% to 34% acetonitrile in water with 1 mL/L formic acid in 50 min, followed by an increase in the percentage acetonitrile to 80% (with 20% water and 1 mL/L formic acid in the acetonitrile and the water) in 3 min, as a column-cleaning step. Between the pre-concentration and analytical columns, an electrospray potential of 3.5 kV was applied directly to the eluent via a solid 0.5 mm platina electrode fitted into a P777 Upchurch microcross. Full scan positive mode FTMS spectra were measured between m/z 380 and 1400 on a LTQ-Orbitrap (Thermo electron, San Jose, CA, USA). MSMS scans of the four most abundant doubly- and triply-charged peaks in the FTMS scan were recorded in data-dependent mode in the linear trap (MSMS threshold  = 5.000).

### Peptide and protein identification

Each run with all MSMS spectra obtained was analyzed with Bioworks 3.3.1 (Thermo electron, San Jose, CA, USA). A maximum of totally 1 differential modification per peptide was set for oxidation of methionines and de-amidation of N and Q. Carboxamidomethylation of cysteines was set as a fixed modification (enzyme = trypsin, maximally 2 missed cleavages, peptide tolerance 10 ppm, fragment ions tolerance 0.5 amu).

A combined protein database was constructed from the human and bovine IPI databases (downloaded as fasta files from ftp://ftp.ebi.ac.uk/pub/databases/IPI/current/ accessed August 2009). A set of 31 protein sequences of common contaminants was added including Trypsin (P00760, bovin), Trypsin (P00761, porcin), Keratin K22E (P35908, human), Keratin K1C9 (P35527, human), Keratin K2C1 (P04264, human), and Keratin K1CI (P35527, human). A decoy database was created by adding the reversed sequences using SequenceReverser from the MaxQuant package [41]. These steps gave a database containing 242906 proteins in total.

The peptide identifications obtained were filtered in Bioworks with four filter criteria: ΔCn >0.08, Xcorr >1.5 for charge state 2+, Xcorr >3.3 for charge state 3+, and Xcorr >3.5 for charge state 4+ [42]. Finally, proteins were displayed based on minimally 2 distinct peptides, an Sf score >1, and a probability <0.5. The false discovery rate (the number of hits against the inverted decoy proteins within filter settings divided by the total number of proteins within filter settings times 100%) was 0%. The function of the identified proteins was checked in the UniProtKB database (http://www.uniprot.org/accessed November 2009).

### Protein quantification

Peak height of peptides belonging to an identified protein was determined using Bioworks. For the host defense proteins, the 3 most abundant peptides per protein were summed [43]. The same 3 peptides were chosen for the five replicates. The summed peptide heights were compared between the human and bovine samples using an independent two-sample t-test, using PASW statistics 17 (SPSS Inc, Chicago, IL, USA). If a protein was not detected in a specific sample, the value for the peak height was set to 10^4^ (minimum detection level) for statistical calculations and “<1” in Table 3.
